# The Local Treatment of Psoriasis

**Published:** 1893-09

**Authors:** R. C. Longfellow

**Affiliations:** Cincinnati


### THE LOCAL TREATMENT OF PSORIASIS.*

BY R. C. LONGFELLOW, M. D.,
CINCINNATI.

Psoriasis stands in the front rank of those skin diseases whose course and duration is often chronic, accompaniened by frequent relapses. This disease is one that often exhausts the medical man's storehouse of remedies, as well as his ability to keep the patient satisfied with his condition and treament prescribed. One of the obstacles to proper local treatment is the usual objection of the patient to the remedies employed, their odor, staining qualities or ointment consistence. The essayist has no lauded specifics to offer

*Abstract of paper read before the Dermatological Section of the American Medical Association, Milwaukee, Wis., June 7, 1893.

whose perfume or color charm the patient's nose or eyes, but desires to call out discussion on the few remedies which have been of any special value. Can these few remedies, whose objectionable features have been a vexed question, be dressed in new clothes that will give a less objectionable treatment to the patient? Can we use more agreeable remedies than the ones referred to and hope to secure any favorable results or permanent cure? While this question is of lesser importance in hospital practice, to treat this disease successfully in private patients yet not render them obnoxious to themselves or friends is an important question. Some patients can bear with the scales, keep from his friends the knowledge of his infirmity, yet the remedies prescribed by his medical adviser render him an object of inquiry. The object of local treatment is to macerate the scales, produce desquamation, thus bringing to the surface the hyperemic and diseased skin. In simple cases this can be done by hot baths, plain or medicated, gentle friction, bland oils or ointments rubbed in daily. These applications are allowed to remain for a few days, then the skin is washed in a hot bath, with very mild friction by a cloth or brush. If the scales and patches are not large or extensive they will yield to the simple treatment; with free use of diaphoretics, covering the patches with oiled paper, the maceration and desquamation will be sufficient. In the simple cases the essayist has had excellent results with the daily use of hot bath, a toilet soap composed of green soap seventy-five parts, washed sulphur twenty-five parts, oil rosemary one part. This soap lathers freely, gives a very cleansing and refreshing bath, exerting the desired effect on the scales. One of the obstacles to satisfactory local treatment is to get the patient to remain the proper length of time in the hot bath, which is invaluable in softening and removing the scales. The vaunted springs that have been supposed to be specific in psoriasis can exert no special effect on the disease itself, but the regulation of diet, beverages, food, exercise, long baths, copious drinking of the water, produces the improvement ascribed to the springs. If the patient at home would follow his physician's orders as faithfully and willingly as he does the printed rules of the springs, his reward would be greater improvement and more permanent results. No better method of removing the scales in chronic or severe cases than green soap thoroughly rubbed on each affected patch, and allowed to remain and dry. Such applications should be made every morning for a week ; if possible keep the patient well covered in bed during these applications—give diaphoretics. In three to five days considerable desquamation will occur; after a week the patient should receive a hot bath, gentle friction being used, the remaining scales will be removed, exposing the hyperemic skin beneath. When the patient will not submit to going to bed, or is compelled to be at his daily duties, cannot use green soap, as indicated, a ten per cent. solution of green soap in collodion can be painted on the patches twice daily. Such a solution can be made by first rendering the green soap anhydrous, dissolving in smallest quantity of absolute alcohol, then adding the collodion. This method is more agreeable to patients, being a trifle less active than when soap alone is used, but the same result is accomplished. This method of using green soap prevents it from being rubbed off, soiling the linen, as the patient engages in his daily occupation. The collodion gives relief from itching, nor does the crust produce itching when several layers have been painted on and hardened. The irritation that green soap produces on some tender skins is overcome by the collodion. These applications are made twice daily for a week, then the loose dry crusts are removed, the remaining ones loosening and coming off by the hot soap bath, gentle friction being used by cloth or hands. When the scales have been removed the hyperemic skin must be treated by a remedy that will have a curative effect. Many remedies have been suggested and used without any special degree of satisfaction. During the few minutes allotted to this subject only the three remedies will be mentioned which have been of real service and most used at the present time, namely, pix liquida, chrysarobin and acidum pyrogallicum. These are disagreeable agents in the matter of odor, staining qualities and ointment consistence when prescribed in the usual way. In some patients the use of tar produces a febrile condition, the skin is irritated and looks as though a dermatitis would supervene. The method of using tar which is least objectionable, the essayist has found, is to make a fifteen per cent. solution in collodion. Each patch is painted every other day until four or five applications have been made, then the layers of collodion being removed the patient is given a hot bath, after which the tar is painted on as before. The odor of tar is greatly reduced in the collodion, and has the advantage over other methods in not being rubbed off or soiling the linen, yet loses none of its therapeutic effects. The irritation that tar sometimes produces is overcome by the local anesthetic effect of the collodion. Chrysarobin should be applied with caution and not allowed to extend beyond the area of the infected patch. It stains both skin and linen a yellowish color; frequently, even with caution, will produce considerable irritation, or may have to be suspended. Applied once daily with care, the patches desquamate, the skin becomes of normal thickness and color, contrasting with the brownish color at the limit of former patch. The method of Fox, to use chrysarobin combined with collodion, is the safest and least objectionable of the present modes of application. Affected patches should be painted once daily, or every two days, care being taken to limit its application only to the patch, as even when used most carefully troublesome irritation occurs in not a few cases. These applications made for a week should always be followed by a prolonged hot soap bath, while the general baths of non-affected parts should not be neglected. Pyrogallic acid, less active than chrysarobin, does not produce as much irritation of the skin as the latter remedy. In psoriasis of the head and face pyrogallic acid is much better adapted for local treatment than chrysarobin, but if allowed to come in contact with the hair will stain. Too extensive or careless application of this agent has caused it to be absorbed, resulting in strangury, vertigo and vomiting. The most safe and least objectionable of local applications to head and face are salicylic acid in plaster or collodion, resorcin or aristol in ointment of roses.

